# The complete mitochondrial genome of Taiwan slug-eating snake (*Pareas formosensis*) and phylogenetic analysis

**DOI:** 10.1080/23802359.2021.1920499

**Published:** 2021-10-23

**Authors:** Yan-Lin Liu, Ya-Jun Zhang, Xian Yang, Xiao-Xiang Yang, Zhi-Hua Lin, Li Ma

**Affiliations:** Laboratory of Amphibian Diversity Investigation, College of Ecology, Lishui University, Lishui, Zhejiang, China

**Keywords:** *Pareas formosensis*, mitochondrial genome, phylogenetic analysis, next-generation sequencing

## Abstract

We report the complete mitochondrial genome (mtDNA) of *Pareas formosensis* (Squamata: Colubridae). This circular mtDNA is 17,703 bp in size and consists of 13 protein-coding genes, 22 transfer RNAs, 2 ribosomal RNAs, and 2 non-coding sequence (D-loop). The total of mtDNA was composition of 57.26% A + T and 42.74% G + C (T: 25.21%, C: 28.84%, A: 32.05%, G: 13.90%). The phylogenetic analysis revealed that *P. formosensis* formed a clade with other species of *Pareas*. This mtDNA sequence of *P. formosensis* provides useful data for studying the population genetics and phylogeography of Colubridae.

The Pareatidae was divided into three genera: *Aplopeltura*, *Asthenodipsas* and *Pareas* (Guo and Zhang 2015). *Pareas* is the largest genus, which contains 20 species (Bhosale et al. 2020). *Pareas formosensis* (Van Denburgh 1909) is widespread in South China (Anhui, Jiangsu, Jiangxi, Zhejiang, Fujian, Sichuan, Guizhou, Yunnan, Guangxi, Guangdong, Taiwan) (Zhao 2006). In this genus, the complete mitochondrial genome (mtDNA) was described only in *P. boulengeri* (Huang et al. 2020). Here, we determined the mtDNA of *P. formosensis* in order to provide useful data for studying the population genetics and phylogeography of Colubridae.

The specimen (species voucher: LSU2020MLTWDT01) was collected in Baiyunshan, from Lishui, Zhejiang Province, China (N28.498097°, E119.920808°), then placed in 90% ethanol and stored in Laboratory of Amphibian Diversity Investigation (Contact person: Li Ma, E-mail: lmahz2011@163.com) at Lishui University, China. The total DNA was extracted from muscles using the EasyPure genomic DNA kit (Trans Gen Biotech Co., Beijing, China) from the muscle tissue of *P. formosensis*, and then was sequenced by a sequencing company (Novogene Bioinformatics Technology Co. Ltd., Tianjin, China). We used MITOS web server to carry out and annotate the gene sequence (Matthias et al. 2013). The mtDNA of *P. formosensis* is a closed-circular molecule of 17,703 bp in length. The complete mtDNA of *P. formosensis* (Genbank accession No MW531674) contains 13 protein-coding genes (PCGs), 22 tRNAs and 2 rRNAs, two D-loop and a L-chain replication-initiating non-coding region (NCR). The gene sequence of *P. formosensis* is approximately the same as *P. boulengeri* (Huang et al. 2020). 13 PCGs include *NAD1-6*, *COX1-3*, *ATP6*, *ATP8*, *NAD4L*, and *CYTB*, among which *NAD5* is the longest (1,788bp) and *ATP8* is the shortest (162 bp). The *ND6* subunit gene and eight tRNAs (*tRNA^Gln^*, *tRNA^Ala^*, *tRNA^Asn^*, *tRNA^Cys^*, *tRNA^Tyr^*, *tRNA^Ser(UCN)^*, *tRNA^Pro^*, and *tRNA^Glu^*) were encoded on the L-strand, whereas the other genes were encoded on the H-strand. All PCGS start with an ATG codon except *ND3* begins with ATA codon, and *COX1* starts with GTG. Six genes (*NAD1*, *NAD3-4*, *CYTB*, and *COX2-3*) end with incomplete stop codons (T–/TA-), and four genes (*NAD5*, *ATP6*, *ATP8*, and *NAD4L*, and *CYTB*) end with TAA, *COX1* and *NAD6* end with AGA and *NAD2* end with TAG. The overall base composition for mtDNA sequence of *P. formosensis i*s as follows: T (25.21%), C (28.84%), A (32.05%) and G (13.90%).

Based on the *CYTB* (1086 bp) and *NAD4* (663 bp) with *Asthenodipsas malaccanus* as outgroup by using Bayesian inference (BI) methods in MrBayes v3.2.2 (Figure 1), phylogenetic analyses of *P. formosensis* and other 14 species of *Pareas* were conducted. We used MrModelTest 2.3 to find the best-fit substitution model (GTR + I + G) (Nylander 2004). Phylogenetic analysis revealed that *P. formosensis* was more closely related to *P. hampton* than other species. *P. carinatus* and *P. menglaensis* has the furthest relationship with *P. formosensis*. The result is consistent with previously reported (You et al. 2015; Wang et al. 2020). Essentially, it is useful for subsequent research about the population genetics and phylogeography of Colubridae.

**Figure 1.**
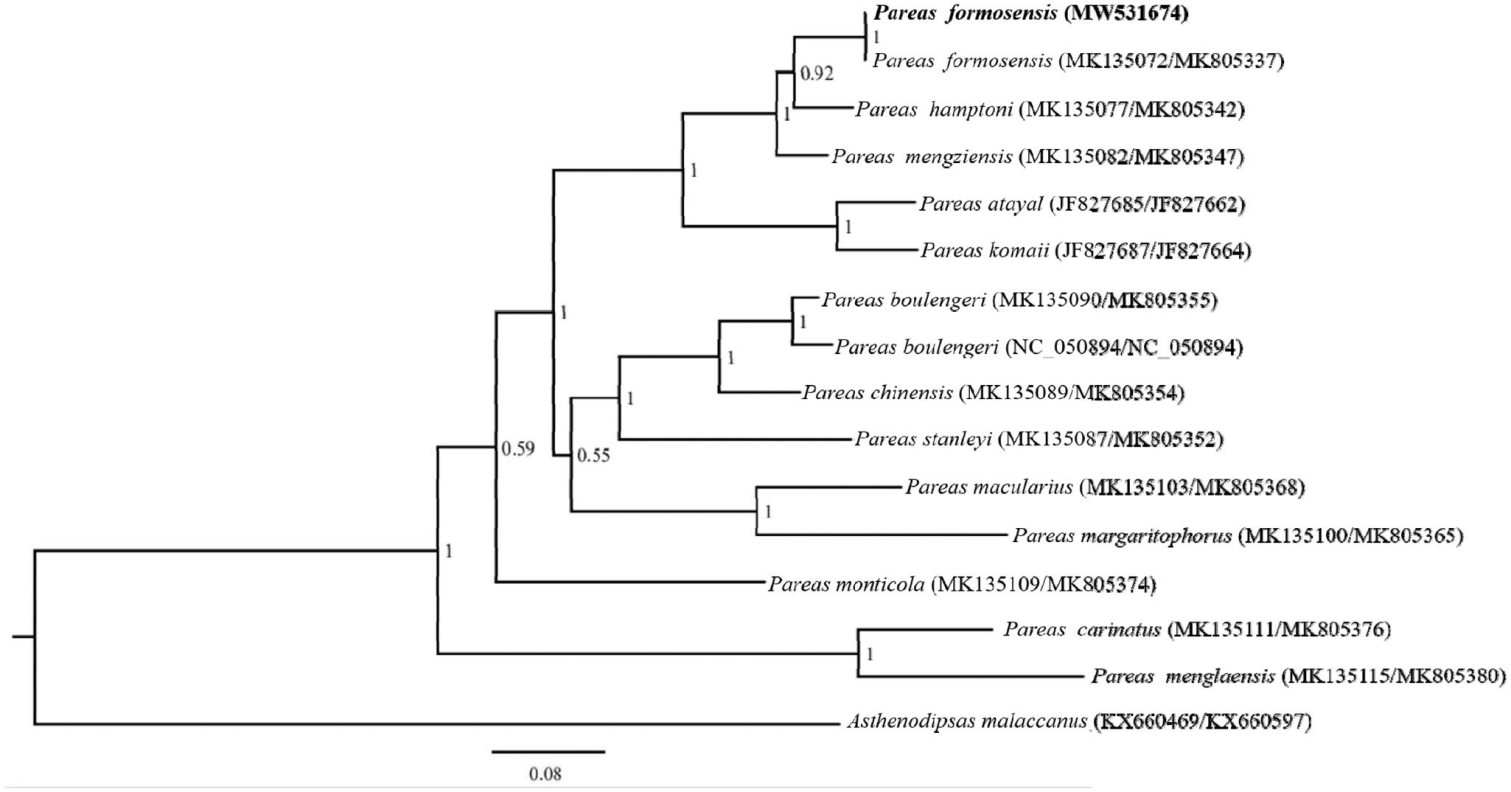
The phylogenetic tree were analyzed with Bayesian inference (BI) method, based on *CYTB* and *NAD4*. The GenBank accession number of *CYTB* and *NDA4* are listed in the figure (*CYTB*/*NDA4*).

## Data Availability

The mitogenome data supporting this study are openly available in GenBank at [https://www.ncbi.nlm.nih.gov/nuccore/ MW531674]. Reference number [Accession number: MW531674]. BioSample and SRA accession numbers are [https://www.ncbi.nlm.nih.gov/biosample/SAMN17394016], [https://www.ncbi.nlm.nih.gov/sra/SRR13495178], respectively.
